# The Medical Profession and Advertising

**Published:** 1881-03

**Authors:** 


### Editorial.

The extensive enterprise displayed in advertising recently by both commercial and educational corporations is very interesting to observe. This interest possesses a separate importance to the medical profession, since they are the medium through which the more subtle and *progressive* advertising measures are projected. We no sooner pass through an era in which certain educational institutions exhibit strenuous, and *apparently* disinterested efforts to establish various educational improvements, and with becoming and commendable sacrifice give the prestige of their name, *prominently*, to the measure; than we enter actively into a season where commercial enterprise and energy are benevolently and gratuitously given to elevate the (medical) professional standard, which is trailing in the dust!

The moves recently made in which it is proposed to protect the poor doctor against the apothecary are loading the mails with matter and crowding the editors' table with trash. Good opportunity is given for mercenary advertising, and the main benefit, as we consider, will accrue to the last-named class.

The principles upon which, ostensibly, the effort is based, appear painfully insufficient to sustain the crusade that has been inaugurated. The innocent (?) little prescription is one great bone of contention, and the ingenuity of each party is taxed to produce conclusive arguments in favor of the right of possession in either party.

Much concern is manifested again, in regard to the prosperity and success of the nostrum venders. Measures are suggested which are calculated to interfere with the promulgation and sale of patent medicines, and to those who will sell them it is advised to put them in a place where they can not be seen! Druggists, who being compelled to keep nostrums for a certain class of trade, may, by the principles of this crusade, readily retain the patronage of the "regular doctors" simply by hiding their "irregular medicines" behind glass!

From Philadelphia emanates the *prospect* of this very desirable innovation. The physicians and druggists of that place are exerting themselves to put the matter fully before their respective professions. There, probably, can be no question of the sincerity of many of the projectors, and to these we would be glad could we offer encouragement. But on the plan they have adopted success is impossible. If the medical profession is to have dignity and honor, it certainly is not to emanate from a competition with druggists and nostrum makers. From a much higher source must we expect to derive prestige and protection. Let the work of reformation begin at home. Let the antagonizing elements and principles of our own organization be abrogated, and in their stead create a unanimity and fraternal friendship, substantial and inviolate. Once this state of things is established, we may consistently direct our energy to the establishment of a social and judicial standard at par with any cotemporaneous profession.

